# Generation of a pancreatic cancer model using a Pdx1-Flp recombinase knock-in allele

**DOI:** 10.1371/journal.pone.0184984

**Published:** 2017-09-21

**Authors:** Jinghai Wu, Xin Liu, Sunayana G. Nayak, Jason R. Pitarresi, Maria C. Cuitiño, Lianbo Yu, Blake E. Hildreth, Katie A. Thies, Daniel J. Schilling, Soledad A. Fernandez, Gustavo Leone, Michael C. Ostrowski

**Affiliations:** 1 Comprehensive Cancer Center, The Ohio State University, Columbus, Ohio, United States of America; 2 Cancer Biology and Genetics Department, The Ohio State University, Columbus, Ohio, United States of America; 3 Center for Biostatistics, Department of Biomedical Informatics, The Ohio State University, Columbus, Ohio, United States of America; 4 Hollings Cancer Center, Medical University of South Carolina, Charleston, South Carolina, United States of America; 5 Department of Biochemistry and Molecular Biology, Medical University of South Carolina, Charleston, South Carolina, United States of America; Centro Nacional de Investigaciones Oncologicas, SPAIN

## Abstract

The contribution of the tumor microenvironment to the development of pancreatic adenocarcinoma (PDAC) is unclear. The *LSL-Kras*^*G12D/+*^;*LSL-p53*^*R172H/+*^;*Pdx-1-Cre* (KPC) tumor model, which is widely utilized to faithfully recapitulate human pancreatic cancer, depends on Cre-mediated recombination in the epithelial lineage to drive tumorigenesis. Therefore, specific Cre-loxP recombination in stromal cells cannot be applied in this model, limiting the *in vivo* investigation of stromal genetics in tumor initiation and progression. To address this issue, we generated a new *Pdx1FlpO* knock-in mouse line, which represents the first mouse model to physiologically express FlpO recombinase in pancreatic epithelial cells. This mouse specifically recombines Frt loci in pancreatic epithelial cells, including acinar, ductal, and islet cells. When combined with the *Frt-STOP-Frt Kras*^*G12D*^ and *p53*^*Frt*^ mouse lines, simultaneous Pdx1FlpO activation of mutant Kras and deletion of p53 results in the spectrum of pathologic changes seen in PDAC, including PanIN lesions and ductal carcinoma. Combination of this KPF mouse model with any stroma-specific Cre can be used to conditionally modify target genes of interest. This will provide an excellent *in vivo* tool to study the roles of genes in different cell types and multiple cell compartments within the pancreatic tumor microenvironment.

## Introduction

Conditional gene knockout is a powerful tool to study the role of individual genes in living organisms. This technique eliminates many of the side effects associated with conventional gene knockout, such as embryonic lethality and the lack of tissue specificity, among others. A conditional knockout approach controls excision of the endogenous target DNA through site-specific recombination (SSR), which involves a recombinase and its short DNA recognition site. The most commonly used site-specific recombinases include Cre (of bacteriophage P1), Flp (of the yeast Saccharomyces cerevisiae) and Dre (of bacteriophage D6). These recombinase enzymes recognize 34 bp Loxp, 34 bp Frt, and 32 bp Rox target sites, respectively, and catalyze a reciprocal conservative recombination between two identical target sequences. This recombination results in a sequence deletion between the two target sites. Although initial studies showed that Cre and Dre were more efficient recombinases than Flp in mammalian cells, a codon optimized version of Flp (termed FlpO), has greatly improved its reliability [1, 2]. Because Cre, Flp, or Dre recombinases are not expressed in mammalian cells, there is no risk of accidental target site recombination in conditional gene knockout mice. Therefore, conditional gene knockout mice are often used as models of human diseases. It has also tremendously increased our ability to study complex human diseases in specific developmental stages and tissues through spatial or temporal gene inactivation via DNA excision. In addition, through combination of different SSR systems within the same model, we can achieve spatiotemporal control of distinct genetic ablation/induction because of independent regulation of distinct recombinases.

PDAC is among the most deadly malignant solid tumors, with over 95% of patients succumbing to the disease within five years of diagnosis, which is largely the result of no effective therapies beyond surgery [3, 4]. The most prominent histopathologic hallmark of pancreatic cancer is its uniquely dense stromal reaction, which consists of activated fibroblasts, increased amounts of extra-cellular matrix (ECM), immune cell infiltrates, and abnormal angiogenesis [5]. The stroma undergoes a dramatic expansion in concert with the step-wise development of PDAC, suggesting that the stroma is an active player in PDAC progression. This has sparked interest in selectively targeting the tumor stroma to increase therapeutic efficacy. However, current knowledge of how the stroma influences tumor initiation, growth, and metastasis remains rudimentary. While current mouse models such as the *LSL-Kras*^*G12D/+*^;*LSL-p53*^*R172H/+*^;*Pdx-1-Cre* (KPC) model accurately reflect the genetics of pancreatic tumor cells and human tumor progression [6–8], these models depend on epithelial-specific Cre-mediated recombination to drive tumorigenesis. Therefore, concurrent Cre-loxP recombination in stromal cells is not practical in these models, limiting the *in vivo* study of stromal genetics in tumor initiation and progression. Thus, the development of mouse models that incorporate stroma-specific genetic modifications in an autochthonous tumor system is crucial to the advancement of the PDAC field. To address this issue, the ideal strategy would be to combine an alternative recombinase system in epithelial cells with Cre-loxP in stromal cells, since *Frt-STOP-Frt(FSF) Kras*^*G12D*^ and *p53*^*frt/frt*^ animals are available. Therefore, we set to develop a knock-in mouse model termed as *Pdx1FlpO*, in which the FlpO gene is specifically inserted into the transcriptional start site of pancreas-duodenum homeobox 1 (Pdx1). We then combined this Pdx1FlpO mice with the above mentioned lines to generate a Flp/Frt PDAC mouse model, *Pdx1FlpO*^*ki*^;*FSF-Kras*^*G12D/+*^,*p53*^*frt/frt*^ (KPF), which is comparable to the historical KPC tumor model.

A transgenic *PdxFlpO* mouse model was recently reported [9]. However, two lines of these *PdxFlpO* mice showed the transgene located in chromosome 1 and 12, while the normal physiologic location of the Pdx1 gene is chromosome 5. With these different chromosomal integration sites, FlpO expression may be influenced by transcriptional regulators other than authentic pancreas-specific regulators. Therefore, the effects of FlpO recombination on mutant Kras and p53 activation may have off-target effects other than normal Pdx1 residential tissues (pancreas, duodenum, and bile duct).

The results presented herein demonstrate the exclusive expression of FlpO in Pdx1 expressing tissues in our knock-in *PdxFlpO* mouse model. FlpO is only expressed in duodenal epithelium, pancreatic acinar cells, ductal cells, and islet cells with no expression in the stroma. This provides an excellent model to investigate genes of interest specifically in stromal cells when combined with stroma-specific Cre-loxP. The knock-in p53 heterozygous and homozygous mice (KPF) develop the spectrum of pathologic changes seen in human PDAC, including acinar to ductal metaplasia (ADM), pancreatic intraepithelial neoplasia (PanIN) and invasive ductal carcinoma, with average survival times of 4.5 and 2 months, respectively. Moreover, the variability of the average survival times is small when compared to published transgenic KPF models, indicating a highly reliable and reproducible KPF model. In summary, this newly developed knock-in KPF mouse model is a valuable tool for studying stromal tissue-specific function of genes of interest in the pancreatic tumor microenvironment.

## Results

### Generation of Pdx1FlpO^ki^ mouse line

**Fig 1:**
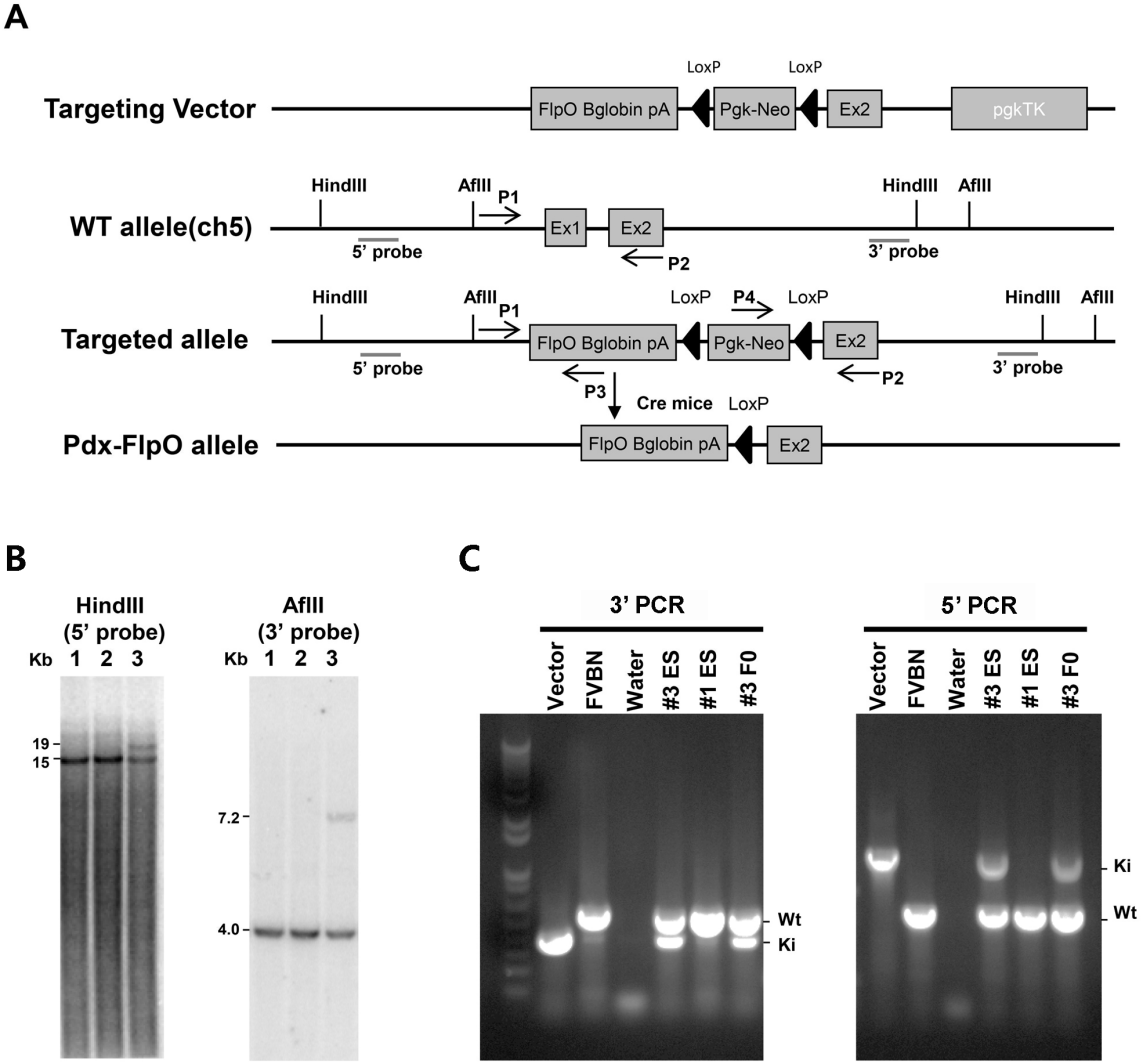
Generation of *Pdx1FlpO*^*ki*^ mice. (A) Schematic representation of the targeting vector, the wild-type Pdx1 locus, the Pdx1-FlpO/beta globin polyA/LoxP-Neo^R^-LoxP targeting allele, and the Pdx1FlpO^ki^ allele after removal of LNL cassette. Mice containing the Pdx1FlpO^Pgk-NeoR^ allele were bred with Sox2-Cre-expressing transgenic mice to remove the LoxP-flanked NeoR cassette. Restriction sites: HindIII and AflII. Primer locations: p1, p2, p3, and p4. A Pgk-TX cassette was placed following exon 2 as a negative selectable marker. (B) Southen blot analysis: Genomic DNA from the aforementioned ES cells was digested by either HindIII or AflII, and hybridized with DNA probes that bind to either 5’ or 3’ of Pdx1 locus. (C) PCR analysis on both the 5’ (p1 and p3) and 3’ (p2 and p4) ends of the targeting vector, WT FVB/N mouse tail DNA, ES cell clone #3, ES cell clone #1, and tail DNA from the F0 chimera mouse generated from ES cell clone #3.

Codon usage-optimized Flp (FlpO) cDNA linked with beta-globin polyA was fused into the start codon site of the Pdx1 gene, replacing the CFP in the previously generated Pdx1CFP targeting vector [10]. A LoxP-Neo^R^-LoxP (LNL) fragment was introduced to select neomycin resistant clones, while a DNA fragment containing mouse phosphoglycerol kinase promoter (Pgk)-driven expression of thymidine kinase was inserted outside of the short arm for negative selection. Therefore, this targeting allele contained the mouse Pdx1 long homology arms of 8.1kb, FlpO cDNA/beta-globin polyA, flanked neomycin resistant cassette (LNL), Pdx1 short arm of 3.6kb and a Pgk-TK cassette, respectively (Fig 1A). Homologous recombination resulted in replacement of a 4.0 kb region of the Pdx1 locus containing exon 1 with codon usage-optimized FlpO cDNA, human beta-globin polyA, and a Pgk-driven neomycin resistance gene flanked by LoxP sites. Clones of interest that had undergone the expected homologous recombination were identified by a 19.0 kb band after digestion with HindIII after hybridization with a 5’ probe, and a band of 7.2 kb after digestion with AflII hybridized with a 3’ probe (ES cell clone #3, Fig 1B). After microinjection of positive ES cell clone WO3 into blastocysts of C57BL/6 mice, the first generation (F0) of *Pdx1FlpO*^*LNL*^ mice was examined by genotyping PCR using primers targeting allele recombination in both the 5’ and 3’ arms. Correctly recombined clones were identified by 0.4 and 1.1 kb bands on the 5’ and 3’, respectively (Fig 1C). The LoxP-flanked NeoR sequence in *Pdx1FlpO*^*LNL*^ (F0) mice was further removed by breeding to *Sox2-Cre* mice. The resultant *Pdx1FlpO*^*knockin*^ (denoted *PdxFlpO*^*ki*^) allele was maintained in a mixed background.

### *In vivo* expression specificity test of the Pdx1FlpO allele

**Fig 2:**
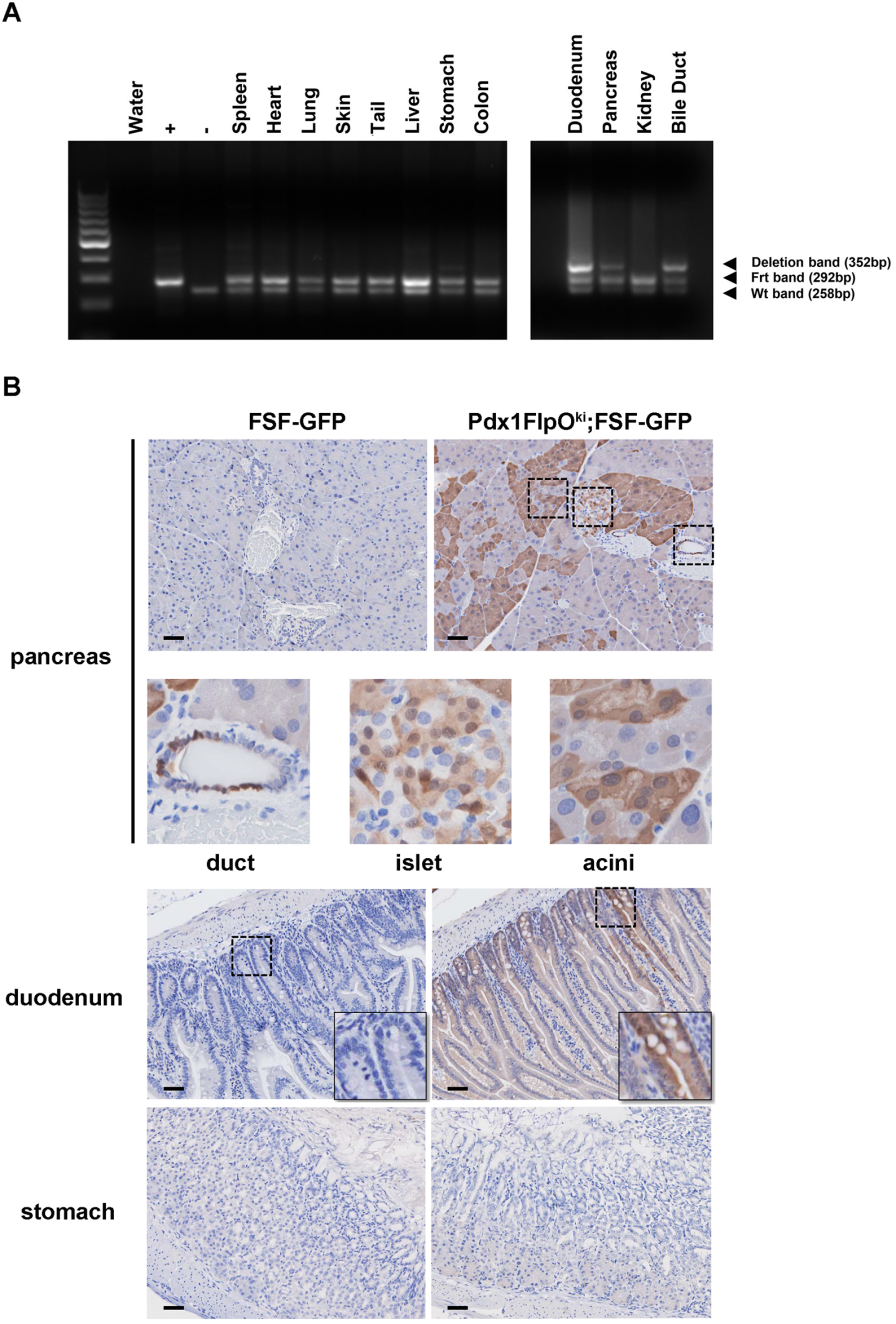
*In vivo* Pdx1FlpO^ki^ allele expression specificity. (A) PCR analysis of Pdx1FlpO^ki^ mediated recombination of the p53Frt allele in the indicated tissues of *Pdx1FlpO*^*ki*^;*p53*^*Frt/+*^ mice. (B) Representative GFP IHC staining demonstrates mosaic GFP expression in the pancreas and duodenum, but not the stomach, of *Pdx1FlpO*^*ki*^;*FSF-GFP* mice. Scale bars = 25 μm.

To evaluate the expression of FlpO recombinase in multiple tissues, we crossed our *Pdx1FlpO*^*ki*^ mouse with the *p53*^*frt/frt*^ mouse, of which exons 2–6 of the p53 gene are flanked by two Frt sites [11]. After collecting all tissues from *Pdx1FlpO*^*ki*^;*p53*^*Frt/+*^ mice, we observed recombination of the p53 Frt allele in the pancreas, duodenum, and bile duct by PCR (Fig 2A). Spleen, heart, lung, skin, liver, stomach, colon, and kidney demonstrated no recombination (Fig 2A).

To further validate this model, we crossed *Pdx1FlpO*^*ki*^ mice with *Frt-stop-Frt (FSF)-GFP* reporter mice. Immunohistochemical (IHC) staining for GFP on tissues isolated from *Pdx1FlpO*^*ki*^;*FSF-GFP* mice revealed GFP expression in the pancreas, duodenum and bile duct but not in the stomach and other tissues (Fig 2B and data not shown). More specifically, GFP expression in the adult pancreas is mosaic and present in a fraction of ductal cells, islet cells, and acinar cells (Fig 2B and Figure A in S1 Fig). This is consistent with the previous reports using *Pdx1-Cre* and *Pdx1-FlpO* transgenic mice. In addition, there was no GFP expression in control *FSF-GFP* mice. Taken together, these data suggest that Pdx1FlpO knockin allele expression is restricted to the pancreas, duodenum, and bile duct.

### *Pdx1FlpO*^*ki*^;*FSF-Kras*^*G12D/+*^;*p53*^*frt/+*^ mice develop locally advanced PDAC and have a significant reduction in survival time

**Fig 3:**
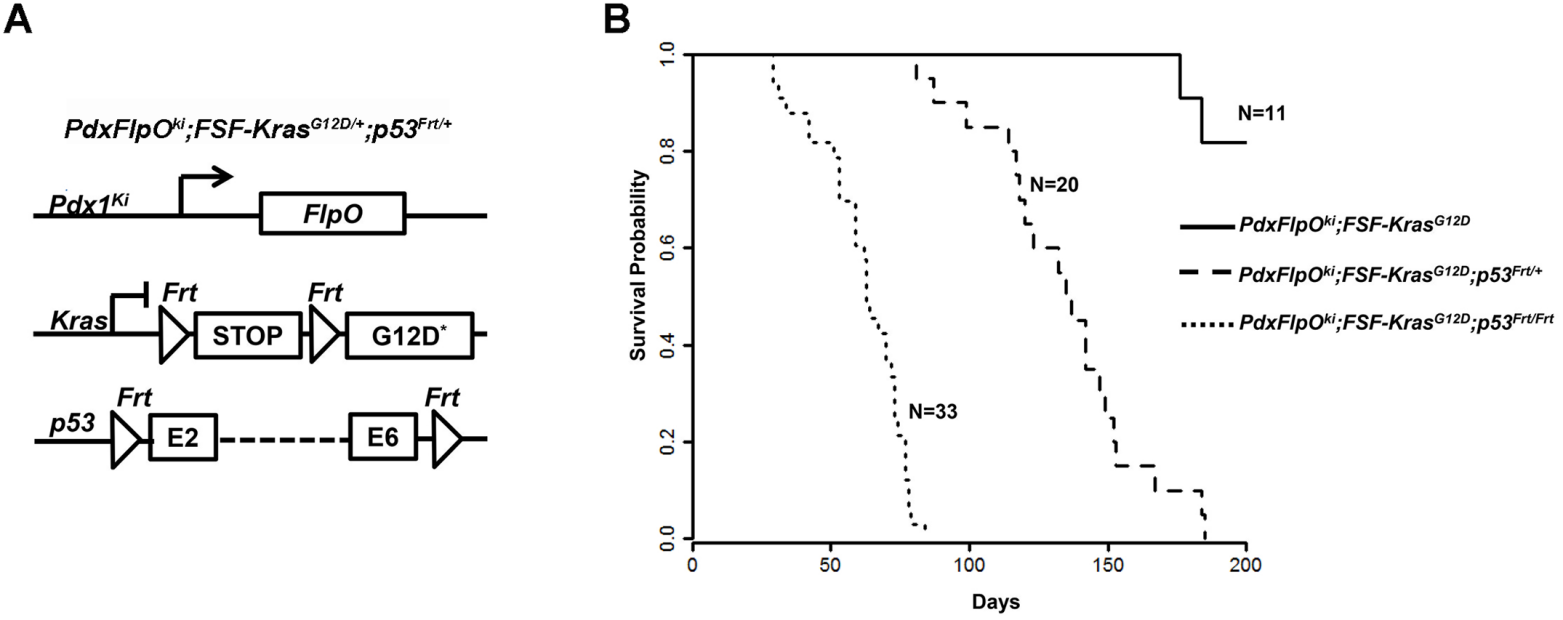
Kaplan-Meier survival with p53 inactivation. (A) Genetic strategy used to generate p53 heterozygous and homozygous deletion in *Pdx1FlpO*^*ki*^;* FSF-Kras*^*G12D*^ mice. (B) Kaplan-Meier survival curves of the indicated genotypes. Median survival of *Pdx1FlpO*^*ki*^;*FSF-Kras*^*G12D/+*^;*p53*^*frt/+*^ or *Pdx1FlpO*^*ki*^;*FSF-Kras*^*G12D/+*^;*p53*^*frt/frt*^ mice is significantly lower than wild-type (WT) mice (p < 0.001, log-rank test for each pairwise combination).

**Fig 4:**
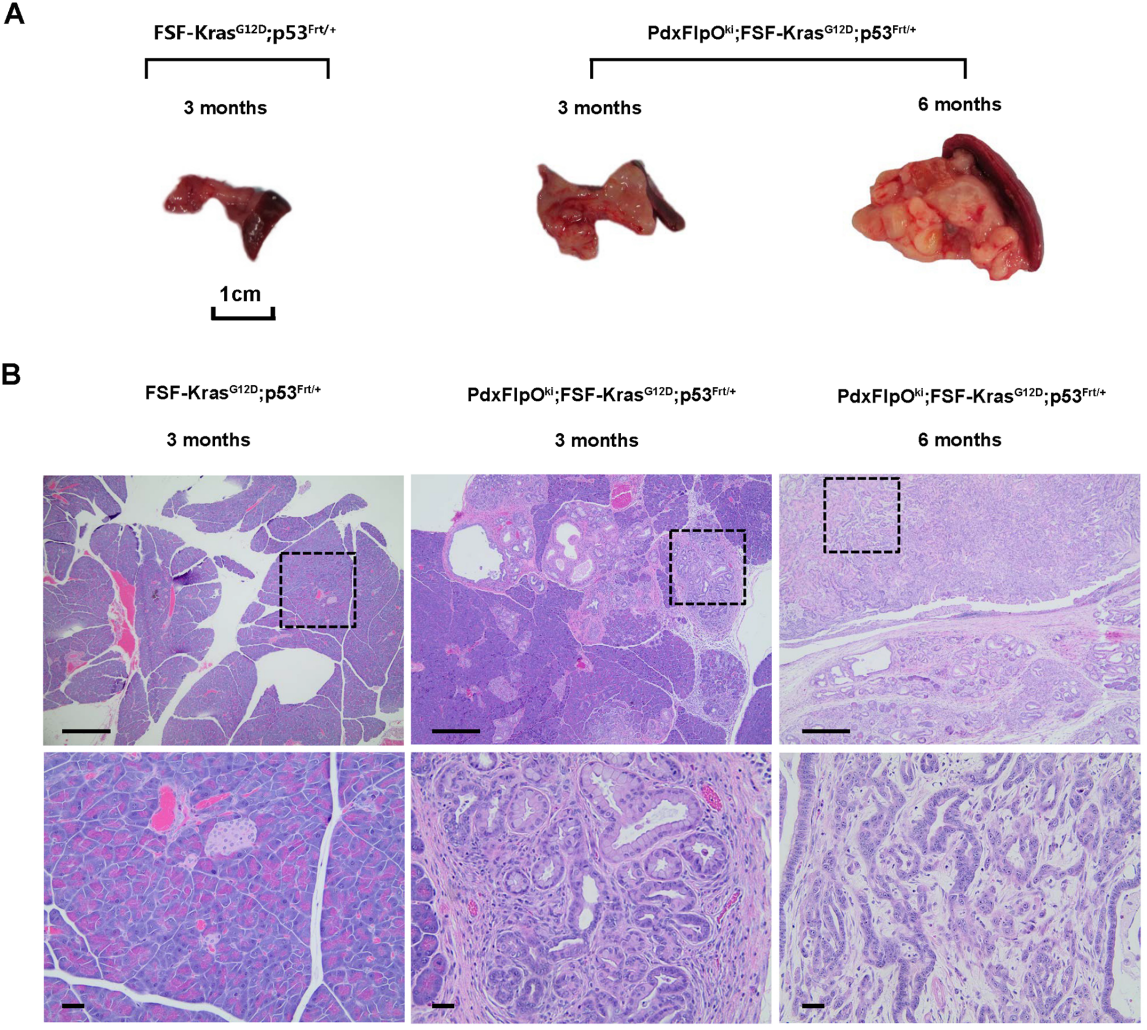
p53 knockout accelerates PDAC formation. (A) Representative macroscopic view of pancreata from *Pdx1FlpO*^*ki*^;*FSF-Kras*^*G12D*^;*p53*^*Frt/+*^ mice at 3 and 6 months of age. (B) Representative microscopic H&E stained pancreatic sections from *Pdx1FlpO*^*ki*^;*FSF-Kras*^*G12D*^;*p53*^*Frt/+*^ mice at 3 and 6 months. Scale bars = 25 μm.

Next, we tried to activate the KrasG12D allele and silence the p53 allele in murine pancreatic progenitor cells by crossing *Pdx1FlpO*^*ki*^, *FSF-Kras*^*G12D*^ and *p53*^*frt*^ animals. To confirm the induction of Pdx1 in early pancreatic lesions, we evaluated Pdx1 expression in 3-month old *Pdx1FlpOki;FSF-Kras*^*G12D/+*^;*p53*^*frt/+*^;*FSF–GFP* mice. IHC staining for Pdx1 in pancreatic tissue revealed robust Pdx1 expression in PanIN lesions (Figure B in S1 Fig), indicating that Pdx1 induction occurs in PanIN lesions in our KPF mouse model. Further, losing one p53 allele, i.e. *Pdx1FlpO*^*ki*^;*FSF-Kras*^*G12D/+*^;*p53*^*frt/+*^, results in a marked decrease in median survival time (~4.5 months) when compared with their control littermates (*Pdx1FlpO*^*ki*^;*FSF-Kras*^*G12D/+*^ animals) (Fig 3A and 3B). This entire cohort of mice (n = 20) showed invasive PDAC at necropsy. Loss of two p53 alleles, i.e. *Pdx1FlpO*^*ki*^;*FSF-Kras*^*G12D/+;*^*p53*^*frt/frt*^, resulted in an even greater decrease in median survival (~2 months). Interestingly, our KPF mouse model showed a significant reduction in survival time when compared to established transgenic KPF models [9], with a median survival of 135 days versus 183 days in the KPF model in P53^frt/+^ animals and a median survival of 60 days versus 85 days in the KPF model in P53^frt/frt^ animals. This likely results from our Pdx1FlpO model possessing a knock-in allele instead of transgenic allele that is present in the KPF model. Histologically, *Pdx1FlpO*^*ki*^;*FSF-Kras*^*G12D/+*^;*p53*^*frt/+*^ mice developed classical PanIN lesions and PDAC. At 2–3 months of age, both low and high grade PanIN lesions were observed, but by 6 months, invasive carcinoma was present (Fig 4A and 4B). The average weight of the pancreas in *Pdx1FlpO*^*ki*^;*FSF-Kras*^*G12D/+*^;*p53*^*frt/+*^ and *Pdx1FlpO*^*ki*^;*FSF-Kras*^*G12D/+*^;*p53*^*frt/frt*^ mice increased significantly compared to *Pdx1FlpO*^*ki*^;*FSF-Kras*^*G12D/+*^ mice (See S1 Table for details). However, unlike previously described transgenic KPC and KPF mouse models, which exhibit metastasis to the liver and lung, we observed negligible metastasis to distant organs, with no lung and only two liver metastases upon evaluation of 53 of our *Pdx1FlpO*^*ki*^;*FSF-Kras*^*G12D/+*^;*p53*^*frt/+*^ and *Pdx1FlpO*^*ki*^;*FSF-Kras*^*G12D/+*^;*p53*^*frt/frt*^ mice combined (Table 1).

**Table 1:** Metastasis spectrum in knock-in KPF mice.

	Total Number	Liver Metastasis	Lung Metastasis
***Pdx1FlpO***^***ki***^**;*FSF-Kras***^***G12D/+***^**;*p53***^***frt/+***^	**20**	**2**	**0**
***Pdx1FlpO***^***ki***^**;*FSF-Kras***^***G12D/+***^**;*p53***^***frt/frt***^	**33**	**0**	**0**

## Discussion

In the last decade, significant advances have been made in understanding the molecular mechanisms underlying human pancreatic cancer using genetically-engineered PDAC mouse models. Compared to xenograft mouse models, these GEM models strongly resemble the histopathology of human PDAC development, providing a great tool to evaluate preclinical therapeutic strategies for the treatment of pancreatic cancer.

All PDAC GEM models are based on Kras activating mutations combined with additional deletions or mutations of other PDAC suppressors such as p53, Smad4, Tgf-β or PTEN [12, 13]. The most widely used and accepted model is the *Pdx1-Cre;LSL-Kras*^*G12D/+*^; *LSL-p53*^*R172H/+*^ transgenic mouse of which mutated Kras and p53 are specifically expressed in pancreatic progenitor cells and are driven by the transcription factor Pdx1. However, due to the nature of Cre-LoxP technology, this model is limited in regards to elucidating the function of other gene targets in different cell types present within the tumor, e.g. in the stromal mesenchymal and immune cell subpopulations. More recently, the generation of KPF mice has provided a solution for this issue. This model uses an alternative Flp/Frt-based recombinase system in the stroma to drive spontaneous PDAC and possesses similar pathology as the KPC mouse model [9]. By combining these two complementary recombinase strategies, gene expression can be manipulated simultaneously in different cell types present within the tumor. This will contribute significantly to the understanding of the relationship between stroma and PDAC progression in a more mechanistic manner.

However, both KPC and existing KPF mouse models are transgenic mice. Compared to a knock-in mouse model which is “specifically targeted”, transgenic models are well known for their “random integration”–the desired gene could end up anywhere in the host genome and with an unpredictable amount of copy numbers inserted. This “randomness” of transgenic models is a limitation due to their unpredictability since the desired gene might be placed under the influence of another strong promoter with a high copy number. Unpredictable levels of overexpression could lead to high variability in the magnitude of the disease [14]. Thus, it might not serve as a reliable and reproducible model of human disease. In addition, studies have demonstrated the leakiness of the previously reported transgenic PdxCre allele in other tissues, most notably in gastric and oral mucosa, which has led to the development of extra-pancreatic tumors such as papillomas and adenomas [7, 14].

In this study, we generated a Pdx1FlpO knock-in mouse line. The Pdx1 gene is expressed in pancreatic progenitor cells starting at embryonic day (E) 8.5. As development proceeds, Pdx1 becomes highly expressed in β cells with lower levels expressed in acinar and other endocrine cells [15]. The mosaic Cre-mediated recombination that occurs within the pancreas using the Pdx1 enhancer/promoter is well established [16]. More importantly, expression of Pdx1-Cre has also been shown in skin keratinocytes [17]. By breeding with the *FSF-GFP* mouse line, we verified that our knock-in *PdxFlpO* demonstrated a similar mosaic expression pattern within acinar, ductal and islet cells in a lineage-specific manner which is consistent between littermates. More importantly, in contrast to Pdx1-Cre, we did not observe Pdx1-FlpO expression in the skin. This finding suggests that the leakiness identified in *Pdx1-Cre* mice might be due to its transgenic targeting strategy.

Compared to the existing transgenic KPF model [9], our knock-in KPF mouse had a decreased survival time. The discrepancies between these two studies could be the timing of FlpO recombinase expression, extension of recombinase expression, genetic background, etc. The timing of FlpO expression in the existing transgenic model was not described [9]. Previous work in the literature demonstrates that the regulatory elements used to generate both PdxCre and PdxFlpO transgenic alleles were sufficient to drive pancreatic expression of Pdx1 alleles with the same developmental timing as the endogenous gene [18], although the location of transgene insertion may affect the timing of expression. Thus, it is difficult to predict when expression began since this transgene was located in Chromosome 12C1-3 instead of the physiological locus of Chromosome 5. In contrast, our knock-in FlpO was located in the normal physiological location of the Pdx1 gene (Chromosome 5). Publications from different labs characterized the expression pattern of Pdx1 *in vivo* using knock-in mouse models [10, 19]. Micallef et al presented a knock-in GFP reporter in the endogenous Pdx1 locus, a strategy similar to our *Pdx1FlpO* model [19]. Their pattern of GFP expression was identical to that reported for the endogenous Pdx1 gene (E9.5, in the dorsal and ventral pancreatic anlage) [20–22], with GFP not being detected in other embryonic tissues. In addition, similar phenotypes were observed in a well-characterized Pdx1CFP mouse line [10], in which CFP was also inserted into exon 1 of the Pdx1 locus by homologous recombination. Both Pdx1 and CFP were detected in the dorsal and ventral endodermal evaginations of the posterior foregut at E9.5. From E10.5 to E11.5, Pdx1 and CFP were detected throughout the pancreatic epithelium. They concluded that this faithfully recapitulated the normal physiologic expression of endogenous Pdx1. Using their knock-in targeting vector Pdx1CFP as a backbone, we successfully replaced the Pdx1 reporter-cyan fluorescent protein (CFP) with FlpO recombinase in our knock-in Pdx1FlpO mouse line. Because the literature already extensively documents the timing of expression of reporter genes knocked into this locus with identical strategies, it is reasonable to predict that the timing of the initiation of FlpO recombinase expression in our mouse model should be the same as it is normally for Pdx1 expression *in vivo*. In addition, in our adult knock-in *Pdx1FlpO* mice, Pdx1-induced FlpO recombination resulted in GFP expression in PanIN lesions. This indicated that Pdx1 induction is present exclusively in the epithelial lesions of our adult KPF tumor model. These findings are consistent with the previous reports using *Pdx1Cre* (7) and *Pdx1FlpO* transgenic mice [9].

PDAC is a cancer type in which mutant p53 impacts disease progression. In addition to the most frequent Kras gene mutation, the p53 gene is also often mutated in human pancreatic cancer in 50–75% of cases [23, 24], predominantly through missense mutations such as R175H and R273H. Consistent with a role for mutant p53 in other mouse models, mice bearing PDAC driven by oncogenic Kras and a mutant p53 allele have shown a greater number of metastases compared to similar mice bearing a p53 null allele [25], likely resulting from up-regulation of PDGFR-β [26]. A full spectrum of PDAC tumorigenesis, including PanINs and carcinoma, were observed in every single mouse in our study cohorts. However, we have detected minimal metastasis to distant organs (2 liver metastases out of 53 mice), with loss of either one or both p53 alleles. This is consistent with other reports on cancer metastasis in mice harboring a p53 null allele, and likely results from ablating the p53 gene instead of activating p53 mutations in the pancreatic epithelium. Of note, there is no *FSF-p53* mutant model available currently. In the future, generation of the FlpO-activated p53 mutant mouse line would greatly benefit this alternative KPF pancreatic cancer model to evaluate metastasis.

Taken together, our knock-in *Pdx1FlpO*^*ki*^ line is more stable and tissue-specific when compared to existing transgenic mouse lines. Thus, it has the potential to contribute to **1)** studies evaluating therapeutics at different stages of tumor progression; **2)** determining the interaction between multiple tumor suppressors and oncogenes; and **3)** the development of new diagnostic approaches. Notably, this *Pdx1FlpO* model is not inducible; therefore, it will be desirable to develop a conditional knock-in model. This latter system would allow investigators to turn on/off genes using this recombinase at discrete time points, such as in adulthood. Future studies should also investigate its compatibility with Cre-loxP targeting of genes of interest in stromal populations.

## Materials and methods

### Pdx1FlpO knock-in targeting

To avoid potential self-recombination by FlpO (Codon usage-optimized Flp), the Frt-hygroR-Frt fragment was initially removed from the Pdx1^CFP+HygroR^ exchange vector [10] by NotI digestion and subsequent re-ligation. A PCR-amplified FlpO fragment from pCAGGs-FlpO [2] was cloned into the ATG start codon site of the Pdx1 gene in the Pdx1^CFP^ vector, followed by insertion of a blunted LoxP-Neo-LoxP (LNL) fragment into the MluI site. A thymidine kinase (TK) cassette was acquired from EcoRI/PvuII digestion of the pLMJ322 vector. The TK fragment was blunted and ligated into the MluI digested Pdx1FlpO-LNL vector, which resulted in the final targeting vector. This targeting vector was named Pdx1FlpO-LNL-TK. The sequence of the full construct (denoted JW9) was confirmed (3730 DNA Analyzer, Applied Biosystems).

Gene targeting was performed following standard protocols by the Genetically Engineered Mouse Modeling facility at The Ohio State University James Comprehensive Cancer Center. In brief, 1 mg of targeting vector linearized with NotI was electroporated into mouse S1B6 (hybrid C57Bl/6-129Sv) ES cells. Following neomycin selection, homologous recombination was verified by Southern blot. A DNA fragment of 19.0 kb that includes most of the long arm, the FlpO cDNA, the Pgk-neo cassette, and the entire short arm was detected using a 5’ probe, while a HindIII-digested fragment of 15 kb from the WT Pdx1 allele was detected in all ES cells. In contrast, an AflII-digested fragment of 7.2 kb from the FlpO knockin allele was detected using a 3’ probe and generated a fragment of 4.8 kb from the WT Pdx1 allele. Germline transmission was achieved following the microinjection of clone WO3 mES cells into C57BL/6 blastocysts. Chimeras were crossed to Black Swiss.

### Other mouse strains and tumor models

*Kras*^*FSF-G12D/+*^ mice were kindly provided by Tyler Jacks. *p53*^*frt/+*^ and *Rosa*^*LSL-FLF-GFP*^ mice have been described previously [9, 11]. Unless otherwise stated, animals were on a mixed C57Bl/6;129Sv;Black Swiss background. *PdxFlpO*^*ki*^, *FSF-Kras*^*G12D/+*^ and *p53*^*frt/+*^ were interbred to obtain PDAC mice (KPF) with activation of oncogenic Kras^G12D^ and deletion of p53 in the pancreatic epithelium. All animal experimental protocols were approved by the Ohio State University Institutional Animal Care and Use Committee. Mice were monitored and weighed weekly. All control mice (*Pdx1FlpO*^*ki*^, *FSF-Kras*^*G12D/+*^) and experimental mice (*Pdx1FlpO*^*ki*^,*FSF-Kras*^*G12D/+*^,*p53*^*frt/+*^ and *Pdx1FlpO*^*ki*^,*FSF-Kras*^*G12D/+*^,*p53*^*frt/frt*^) were euthanized with carbon dioxide (CO2) and cervical dislocation by 6 months of age. Mice were removed sooner when they met Early Removal Criteria (ERC) which included an unkempt hair coat, decreased alertness and mobility, reduced food and water intake, and a 20% loss of body weight if any of these symptoms persisted for more than 24 hrs. The overall health of the animals was monitored by our trained laboratory personnel and the veterinary staff.

### Histologic analysis and immunohistochemistry

Dissected mouse pancreata were fixed in 10% neutral-buffered formalin for 48 hr and then transferred to 70% ethanol. Tissues were processed, embedded in paraffin, cut in 5μm sections on positively charged slides, de-paraffinized, rehydrated, and stained with H&E. For GFP IHC, slides were heated at 65°C for 15 min and after standard de-paraffinization and rehydration, sections were unmasked in 1X Target Retrieval solution (pH = 6.0). Endogenous peroxidases were quenched in 3% H_2_O_2_/PBS for 15 min. Primary antibody incubation was performed overnight (16 hr) at 4°C. Staining for GFP was preformed using an immunoperoxidase technique (Vectastain Elite ABC kit, Vector Labs) and 3,3’-diaminobenzadine followed by counterstaining with Meyer’s hematoxylin. After counterstaining, samples were dehydrated through ethanols and xylenes, mounted and cover slipped. Pdx1 IHC was performed using the Bond RX autostainer (Leica Biosystems Inc.). For this, slides were baked at 65°C for 15 minutes and the automated system performed dewaxing, rehydration, antigen retrieval, blocking, primary antibody incubation, post primary antibody incubation, detection (DAB), and counterstaining using Bond reagents (Leica). The following primary antibodies were used: rabbit anti-GFP (1:600, Abcam) and rabbit anti-Pdx1 (1:500, Abcam). Images were captured with either the PerkinElmer’s Vectra^®^ multispectral slide analysis system (GFP) or Zeiss Imager A.2 equipped with a Zeiss Axiocam 512 color camera (Pdx1). All images were resized and formatted with Adobe Photoshop CS5 software (Adobe Systems Incorporated).

### PCR genotyping

To identify the Pdx1-FlpO knock-in allele, a genotyping strategy was designed including both the 5’ and 3’ ends (Fig 1A). Primer sequences were: P1:5′-GGAGAACTGTCAAAGCGATC-3’; P2:5’-GCAGCCAAGTCCAGACTAGG-3’; P3:5’-CTTAGGGCCGTTCTTGATAG-3’; P4:5’-TCGACTGTGCCTTCTAG TTG-3’. For the 5’ end, the wild type allele amplified by primer pair P1–P2 produces a PCR product of 576 bp whereas the Pdx1-FlpO allele amplified by primer pair P1–P3 gives a 1100 bp product. For the 3’ end, the wild type allele amplified by primer pair P1–P2 produces a PCR product of 576 bp whereas the Pdx1-FlpO allele amplified by primer pair P2–P4 gives a 399 bp product. All PCR reactions were performed using the following conditions: 94°C for 1 min followed by 35 cycles of 94°C for 30 s, 55°C for 30 s, and 72°C 1 min.

## Supporting information

S1 Fig Percentage of recombination in the pancreas and representative Pdx1 immunohistochemistry. (A) The percentage of recombination in the pancreas. The recombination was quantified by percentage of GFP positive cells in three pancreatic epithelial lineages (ductal cells, islet cells and acinar cells), N = 3 and (B) Representative Pdx1 immunohistochemistry staining demonstrates Pdx1 expression in the pancreatic PanIN lesions (Black arrows) of *Pdx1FlpO*^*ki*^;*FSF-Kras*^*G12D/+*^;*p53*^*frt/+*^;*FSF–GFP* mice (scale bar: 50 μm).
(PPTX)

S1 Table Tumor and animal data. (XLSX)
