# Supplementary material for: Generation of a pancreatic cancer model using a Pdx1-Flp recombinase knock-in allele
Source: PLoS One. 2017 Sep 21;12(9):e0184984. doi: 10.1371/journal.pone.0184984 (PMC5608307; doi:10.1371/journal.pone.0184984)
Supplement: S1 Fig — (A) The percentage of recombination in the pancreas. The recombination was quantified by percentage of GFP positive cells in three pancreatic epithelial lineages (ductal cells, islet cells and acinar cells), N = 3 and (B) Representative Pdx1 immunohistochemistry staining demonstrates Pdx1 expression in the pancreatic PanIN lesions (Black arrows) of Pdx1FlpOki;FSF-KrasG12D/+;p53frt/+;FSF–GFP mice (scale bar: 50 μm). (PPTX) [file pone.0184984.s001.pptx]

## Slide 1
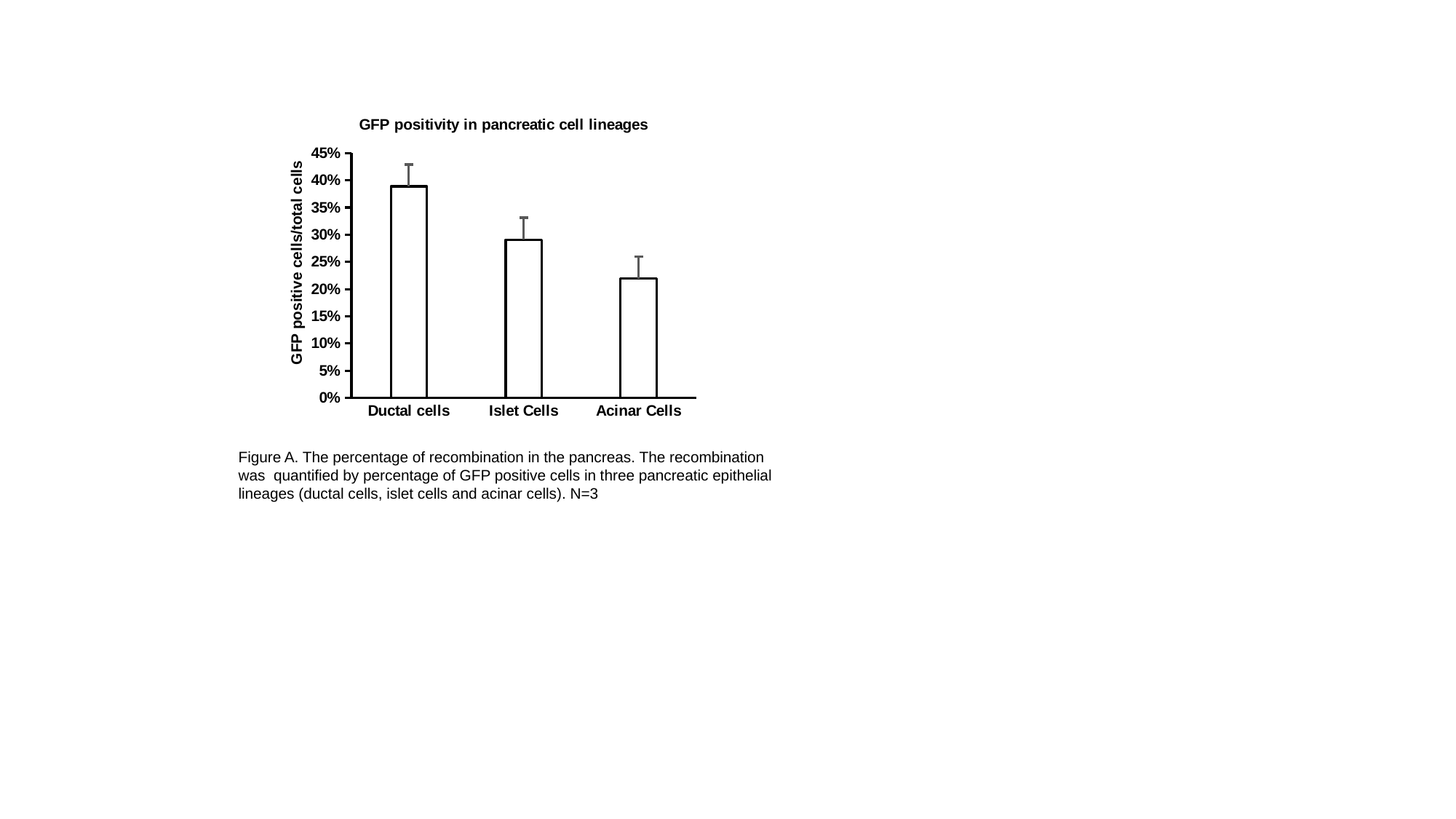

### Chart: GFP positivity in pancreatic cell lineages
| Category | |
|---|---|
| Ductal cells | 0.3889 |
| Islet Cells | 0.2911 |
| Acinar Cells | 0.2192 |GFP positive cells/total cells
Figure A. The percentage of recombination in the pancreas. The recombination was quantified by percentage of GFP positive cells in three pancreatic epithelial lineages (ductal cells, islet cells and acinar cells). N=3

## Slide 2
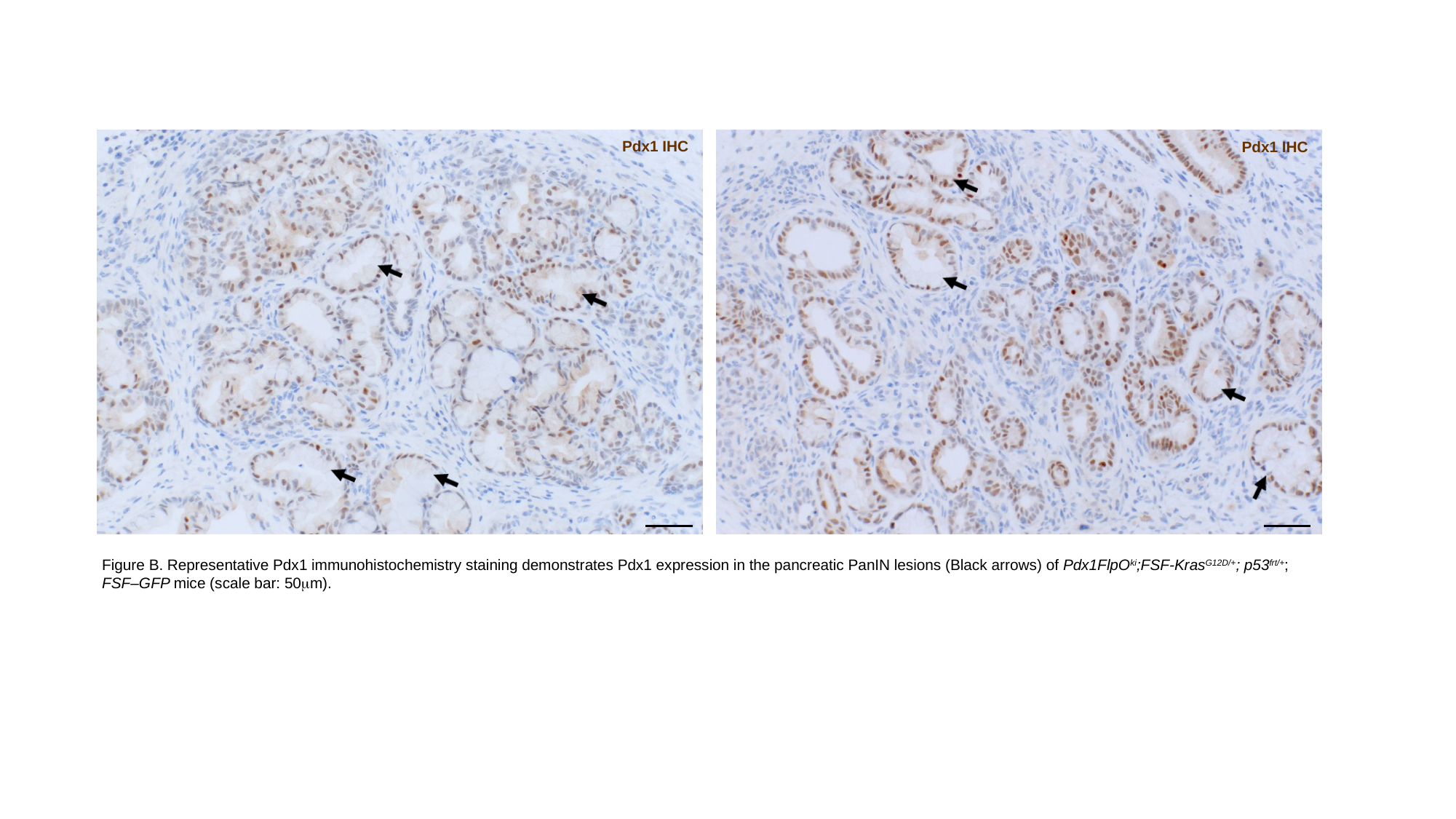

Pdx1 IHC
Pdx1 IHC
Figure B. Representative Pdx1 immunohistochemistry staining demonstrates Pdx1 expression in the pancreatic PanIN lesions (Black arrows) of Pdx1FlpOki;FSF-KrasG12D/+; p53frt/+; FSF–GFP mice (scale bar: 50mm).
